# Sleep, Alzheimer pathology and risk of clinical progression in cognitively unimpaired older adults

**DOI:** 10.1002/alz.095524

**Published:** 2025-01-09

**Authors:** Bery Mohammediyan, Andree‐Ann Baril, Alfonso Fajardo, Judes Poirier, Jean‐Paul Soucy, Sylvia Villeneuve

**Affiliations:** ^1^ McGill University, Montreal, QC Canada; ^2^ Douglas Mental Health University Institute, Centre for Studies on the Prevention of Alzheimer’s Disease (StoP‐AD), Montréal, QC Canada; ^3^ Research Center of the CIUSSS‐NIM, Hôpital du Sacré‐Coeur de Montréal,, Montreal, QC Canada; ^4^ University of Montreal, Montreal, QC Canada; ^5^ Department of Psychiatry, McGill University, Montréal, QC Canada; ^6^ McConnell Brain Imaging Centre ‐ McGill University, Montreal, QC Canada

## Abstract

**Background:**

Increasing evidence suggests a link between sleep and Alzheimer disease’s (AD) pathology and cognitive decline. We investigated whether sleep disturbances might be accompanied by faster AD pathology accumulation and/or cognitive decline before the onset of cognitive symptoms.

**Method:**

We investigated cross‐sectional and longitudinal associations between sleep quality, AD pathology and cognition in 220 participants from the PREVENT‐AD cohort. A subsample of 99 participants had longitudinal amyloid and tau PET data (mean follow‐up:4.33±0.53y, range: 1.59 – 6.11y) and a subsample of 218 had longitudinal cognitive evaluations (mean follow‐up:0.80±0.50y, range 1‐9). We used the PSQI global score and the actigraphy day‐to‐day sleep efficiency and fragmentation index variability, amyloid and tau‐PET to measure amyloid and tau respectively and the Repeated Battery for the Assessment of Neuropsychological Status (RBANS) to assess cognition. All participants were cognitively unimpaired at their first sleep measurement and 32 individuals developed mild cognitive impairment (MCI) during the study. In supplementary analyses individuals were classified as having high or low to moderated levels of amyloid based on a centiloid of 40. We used robust linear models (RLM) and ANOVAs to assess the association between sleep, and AD pathology and cognition.

**Result:**

We found that higher levels of amyloid pathology were associated with greater day‐to‐day sleep efficiency and fragmentation index variability (Fig. 1). Higher levels of tau pathology were associated with greater day‐to‐day sleep efficiency and fragmentation variability (Fig. 1). We further found longitudinal associations between annual amyloid change and greater day‐to‐day sleep efficiency variability (Fig. 1). These associations were only present in individuals who had centiloid values lower than or equal to 40 (Fig. 2). While no association was found between sleep quality and cognition, individuals who developed MCI (n = 32) had higher baseline PSQI scores (Fig. 3) and greater day‐to‐day sleep efficiency (Fig. 3) years before they were classified as MCI.

**Conclusion:**

Higher variability in sleep quality and worst self‐reported sleep relates to AD pathology. Subjective and objective sleep impairments were also present years prior the development of MCI. Sleep variability and subjective sleep assessment changes might precede sleep disruptions observed later in the disease, which could promote further pathological processes in the brain.

